# The Examinations of the Future

**Published:** 1895-10-12

**Authors:** 


### The Hospital

AN INSTITUTIONAL JOURNAL OF

The Medical Sciences and Hospital Administration,

WITH

AN EXTRA NURSING SUPPLEMENT.

VOL. XIX.—No. 472] [OCT. 12, 1895.

### THE EXAMINATIONS OF THE FUTURE.

MR. JONATHAN HUTCHINSON has been a student, a teacher, an examiner, and a practitioner of medicine, and in every one of those capacities he has excelled. At the opening of the winter session of the Liverpool School of Medicine the other day, under the presidency of the Earl of Derby, he was at pains to elaborate views on the subject of medical examinations, all of which were judicious and admirably stated, and some of which were novel and almost revolutionary. The medical profession always hears Mr. Hutchinson with pleasure, and on the present occasion there is no doubt that his views will be very critically weighed by all the best minds among us, whether we be students, teachers, examiners, or practitioners.

It has become a matter of the greatest possible moment that only suitable persons shall be admitted to the status and work of the medical practitioner. It is the examiner who holds the keys of the medical kingdom, and on his fitness for his functions depend the quality and quantity of those who are to form the medical profession of the immediate future. Well may Mr. Hutchinson insist upon the paramount importance to medicine of examiners and examinations.

It is hardly necessary to say that Mr. Hutchinson has a root and branch quarrel with many of the methods of past and present examiners. Being a man of real mental capacity and of very wide culture and experience, it is quite impossible that he should have any kind of patience with the mindless, frivolous, and inconsequent proceedings which some official persons dignify with the name of "methods of examination." To ask "catch" questions, to "pin men down" on subjects in which they are "weak," and to give them no chance of showing what they are made of in subjects in which they are "strong," to let "pass" or "pluck" depend upon the mood or the digestion of the examiner—all these are circumstances which were noted by Mr. Hutchinson, and noted to be denounced and classed with those vulgar abuses of opportunity which better culture and truer dignity will make for ever impossible.

But mere denunciation, however stern and just, would not entitle Mr. Hutchinson's utterances to the prolonged and serious consideration of the whole profession. He makes proposals which are definite and new, and which, if carried out in practice, will, in our judgment, result in a measure of knowledge and culture among all classes of students both broader and deeper than any we have hitherto known. The examinations should be broken up, he contends. Instead of taking four, or even three, wide-reaching subjects at one examination, as is done at present, each really important subject should be examined in separately and by itself. With a five years' curriculum this could well be done. Two years could still be devoted to study without examination; and if three examinations were held in each year nine important subjects could be separately examined in before the termination of the five years of compulsory medical study. Mr. Hutchinson believes, and every experienced teacher and practitioner must agree with him, that although something might be forgotten of earlier studies before the close of the curriculum, yet an amount of knowledge of facts and a clear insight into the fundamental principles of the sciences and arts on which medicine is based would thus be gained, infinitely beyond anything of which examiners have had any previous experience.

The word "revolutionary" might almost be applied to Mr. Hutchinson's most novel proposal. The questions to be asked at all examinations, he insists, should all be prepared beforehand, and not only prepared but published. Every five years a catechism of questions should be elaborated, and no question should be asked at any medical examination which had not previously been published in the quinquennial catechism. The result of that would be, says the old-fashioned examiner, that every student would know the answers to the published questions, and nothing else. Most true; and that is exactly the result desired by Mr. Hutchinson, and by all who have seriously considered the subject. The framing of the catechism of questions would be one of the most important of all the parts of medical education. A limited number of questions properly framed and published would give a definite guidance and precision to medical study which it has not hitherto been found possible to give in any other way. Medical education, as at present conducted, is too wide for either precision or depth. However we conduct it, we may not expect any very profound depth to be obtained during the student period in so wide a range of subjects of study as medicine demands. But precision of thought and knowledge we may expect, and, if that be acquired, depth will come with time and experience. Mr. Hutchinson's proposals, made as they are by a medical philosopher, and one of our ablest practising surgeons—made also after an experience of many years in the four departments of studentship, teaching, examining, and practice—demand, and will receive, the most serious consideration on the part of every man who has at heart the highest interests of the medical profession.

